# Observation of Anomalous Resistance Behavior in Bilayer Graphene

**DOI:** 10.1186/s11671-016-1792-z

**Published:** 2017-01-17

**Authors:** Yanping Liu, Wen Siang Lew, Zongwen Liu

**Affiliations:** 1 Department of Materials Science and Engineering, University of California, Berkeley, CA 94720 USA; 2 School of Physical and Mathematical Sciences, Nanyang Technological University, 21 Nanyang Link, 637371 Singapore, Singapore; 3 School of Chemical and Biomolecular Engineering, The University of Sydney, Sydney, New South Wales (NSW) 2006 Australia

**Keywords:** Bilayer graphene, Interlayer ripple scattering effect, Coulomb scattering effect

## Abstract

Our measurement results have shown that bilayer graphene exhibits an unexpected sharp transition of the resistance value in the temperature region 200~250 K. We argue that this behavior originates from the interlayer ripple scattering effect between the top and bottom ripple graphene layer. The inter-scattering can mimic the Coulomb scattering but is strongly dependent on temperature. The observed behavior is consistent with the theoretical prediction that charged impurities are the dominant scatters in bilayer graphene. The resistance increase with increasing perpendicular magnetic field strongly supports the postulate that magnetic field induces an excitonic gap in bilayer graphene. Our results reveal that the relative change of resistance induced by magnetic field in the bilayer graphene shows an anomalous thermally activated property.

## Background

The electronic properties of monolayer graphene have been extensively studied due to its intriguing energy band structure with linear dispersion around the Dirac point and chirality exhibiting Berry phase of *π* [1]. There is a zero-energy Landau level (LL) with fourfold degeneracy due to interactions between electron spins and valleys in the magnetic field [2–4]. Recently, bilayer graphene became a subject of intense research due to the low-energy Hamiltonian of chiral quasiparticles and a Berry phase of 2*π* [5–8]. It has a double-degeneracy zero-energy Landau level that incorporates two different orbital states with the same energy under an external magnetic field. The bilayer graphene with a Bernal (A-B) configuration loses some features of monolayer graphene and has a unique band structure where the conduction and valence bands are in contact with a nearly quadratic dispersion [5]. In bilayer graphene, a parabolic band structure (*E*_*F*_ = *ℏ* ^2^ *k* ^2^/2m*) with an effective mass m* = 0.037*m*_*e*_, has been calculated by using the interlayer coupling model [9–15]. What makes bilayer graphene an interesting material for study is that the interlayer potential asymmetry can be controlled by an electric field, thus opening an energy gap between the conduction and valence bands [16–18]. Various applications for bilayer graphene are possible due to the fact that its bandgap can be modulated by using an external out-of-plane electric field and chemical doping. There is intensive research on bilayer graphene under the application of a perpendicular electric field. However, experimental reports on magnetic transport properties of bilayer graphene are not as well-studied. Recent theoretical work reports on excitonic condensation and quantum Hall ferromagnetism in bilayer graphene [19]. There are interesting features in bilayer graphene due to its extra twofold orbital degeneracy in the LL spectrum, which results in an eightfold-degenerate LL at zero energy. The scattering mechanism of graphene is currently a subject of intense research and debate. The problem of magneto-transport properties in the presence of Coulomb impurities is still an open research problem. Our understanding of the nature of the disorder and how the mesoscopic ripple effect affects the transport properties still need improvement; hence, a better understanding of the general electric and magnetic transport properties of bilayer graphene is necessary.

In this paper, we have systematically investigated the charge transport properties in bilayer graphene as a function of temperature, magnetic field, and electric field. Our measurement results have shown that bilayer graphene exhibits a semi-metallic *R*-*T* property and an unexpected sharp transition of the resistance value in the temperature region 200~250 K. The longitudinal resistance decreases with increasing temperature and electric field, a behavior that is markedly different from the experimental reports of monolayer graphene. Our results reveal that the energy gap in the bilayer graphene shows an anomalous thermally activated property and increases with $\sqrt{B}$.

## Methods

The bilayer graphene flakes in our study were prepared via mechanical exfoliation techniques from the bulk highly oriented pyrolytic graphite (grade ZYA, SPI Supplies) and transferred onto the surface of a lightly doped silicon substrate covered with a 285-nm-thick layer of thermally grown *SiO*_2_. The doped silicon substrate and *SiO*_2_ were used as back-gate and gate dielectric, respectively. Graphene electrical electrodes were patterned using photolithography techniques. A pair of ohmic Cr/Au (5 nm/100 nm) contacts were deposited via thermal evaporation at a background pressure of 10^−7^ mbar and subsequently lifted off in warm acetone. Electronic transport measurements have been carried out on multiple samples, using PPMS (Quantum Design) with a fixed excitation current of 10 μA. Electrical measurements were performed in the temperature range 2~340 K, and a magnetic field up to 12 T was applied. In order to enhance electrical transport, the sample was cleaned in situ by the magnetic and electric field. Four-terminal electrical measurements were used for transport characterization.

## Results and Discussion

**Fig. 1:**
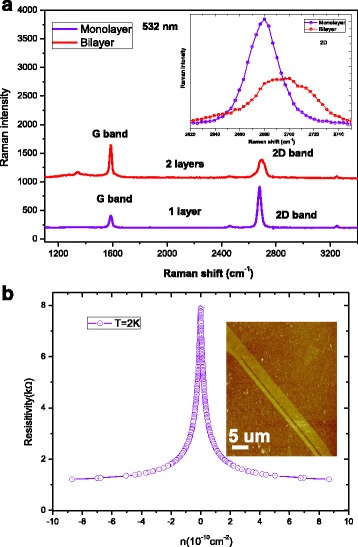
**a** Comparison of Raman spectra at 532 nm for mono- to bilayer graphene. The position of *G* peak and the spectral features of 2D band confirm the number of atomic layer of the graphene. The 2D band (*inset* shows the close-up plot) of bilayer graphene is red-shifted and broadened with respect to that of the monolayer graphene. **b** Four-terminal resistance *R*_xx_ as a function of carrier density *n*

It has been shown that Raman spectroscopy is a reliable, nondestructive tool for identifying the number of graphene layers and it can be done through the 2D band deconvolution procedure [20–22]. The Raman spectra of our graphene structure were measured at room temperature using a WITEC CRM200 instrument at 532-nm excitation wavelength in the backscattering configuration [23–27]. Figure 1a shows the characteristic Raman spectrum with a clearly distinguishable *G* peak and 2D band. The two most intense features are the *G* peak and the 2D band which is sensitive to the number of layers of graphene. The position of the *G* peak and the shape of the 2D band confirm the number of layers of graphene. Additionally, the number of layers of graphene can be easily distinguished from the full-width half maximum of the 2D band, as its mode changes from a narrow and symmetric feature for monolayer graphene to an asymmetric distribution on the high-energy side for bilayer graphene [24]. The 2D band inset in Fig. 1a shows that the Raman spectrum of bilayer graphene is red-shifted and broadened with respect to that of the monolayer graphene. Figure 1b shows the four-terminal resistance *R*_xx_ as a function of carrier density *n*, and the sample shows a pronounced peak at density 5.48 × 10^−10^ cm^−2^. Note that the sharp peak in resistance at low *n* is enhanced by the opening of the small energy gap owing to the disorder-induced differences in carrier density between the top and the bottom layers of the flake.

**Fig. 2:**
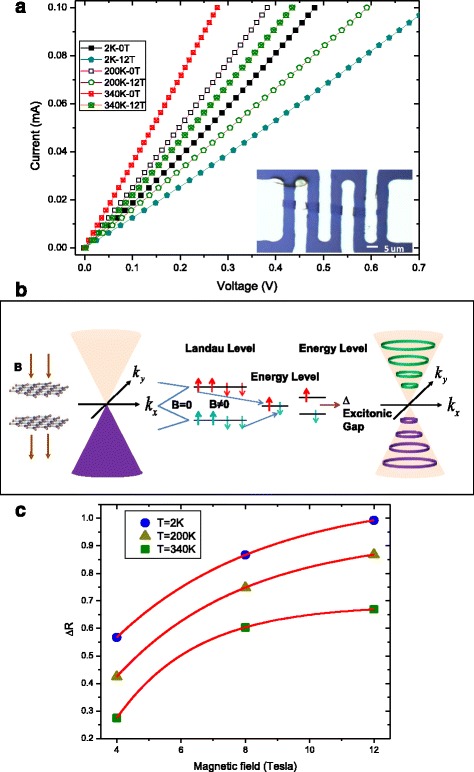
**a** Temperature-dependent current-voltage characteristics of bilayer graphene. The *inset* shows the optical micrograph of the bilayer graphene interconnects with gold electrodes. The measurements show that bilayer graphene intrinsic semiconductor property and the introduction of a perpendicular magnetic field could induce high resistance. **b** Illustrations of bilayer graphene bandgap and Landau level splitting under the application of the magnetic field. The zero-energy state with respect to up-spin electrons and down-spin holes makes an excitation condensation gap due to the attractive Coulomb force between a hole and an electron.**c** The relative change resistance *ΔR* = (*G*_*B* = *Δ*4*T*_ − *G*_*B* = 0*T*_) as a function of magnetic field. *Inset*: The resistance from the inverse of the gradient of the IV curve (Fig. 2b) as a function of the magnetic field at temperature 5, 200, and 340 K

We have characterized the current (*I*)-voltage (*V*) characteristics of the bilayer graphene via four-terminal measurement, at different temperatures and magnetic fields. Shown in Fig. 2a are the *I*-*V* curves for bilayer graphene under the application of various magnetic fields at three different temperatures: 2, 200, and 340 K. The magnetic field is applied in the perpendicular direction to the plane of the graphene. For all the temperatures and magnetic field strengths, the bilayer graphene exhibits a linear *I*-*V* curve. This implies that the graphene layer is ohmic in nature. We observed that for a fixed magnetic field, the I-V curve displays a larger gradient at a higher temperature than at lower temperature. Interestingly, the gradient of the *I*-*V* curve decreases with increasing magnetic field. In our structure, the gradient of the curve corresponds to the conductivity of the graphene layer. Such temperature- and magnetic field-dependent behavior of conductivity are characteristic of an intrinsic semiconductor. The decrease in the conductivity of the bilayer graphene with increasing magnetic field is attributed to the excitonic energy gap induced by the magnetic field. This conductivity dependence on the magnetic field suggests that the resistance $\left(\rho =\frac{1}{\sigma}\right)$ of graphene is a qualitative fingerprint of its bandgap.

In the absence of a magnetic field, the band structure of the bilayer graphene at the Dirac Valley has a parabolic dispersion relation. When a magnetic field is present, the band structure changes to a split Landau level structure [28–30]. Figure 2b is an illustration of the bilayer bandgap and Landau level splitting under the influence of a magnetic field. The inset shows an optical image of the bilayer graphene with the metal contact electrodes. In Fig. 2c, we plot the resistance of the bilayer graphene, as extracted from the *I*-*V* curve, as a function of magnetic field for three different temperatures. As the magnetic field was increased in a step of 4 T, the resistance increase for each step was different, resulting in a nonlinear relationship between the resistance and magnetic field. Interestingly, the observed nonlinear relationship is markedly different from Zeeman spin-splitting theoretical model with the line relationship, where gap∆_z_ = *gμ*_*B*_ *B* with a free-electron *g* factor *g* = 2, where *μ*_*B*_ is the Bohr magneton. This potentially indicates a sublattice symmetry breaking and gap formation due to many-body correction in this LL [31–33]. This is a further confirmation that magnetic field opens an excitonic gap in the bilayer graphene.

**Fig. 3:**
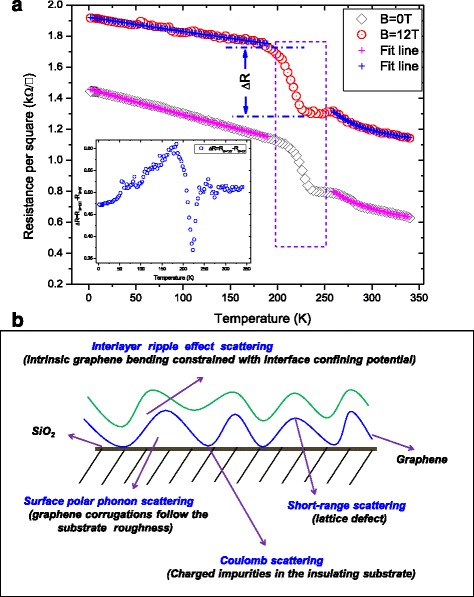
**a** Electrical resistance per square measurements of bilayer graphene as a function of temperature at the different magnetic field (*B* = 0~12 T). The results show that when the temperature increases from 2 to 340 K, the resistance of the bilayer graphene drops significantly. Indicating the bilayer graphene resistors have intrinsic semiconductor properties and magnetic field-induced high resistance behavior in bilayer graphene. *Inset* in **a** shows the relative resistance change under the condition of with and without magnetic field as a function of temperature. The symbols are the measured data, and the lines are fitted. **b** Schematic illustration of scattering mechanisms in bilayer graphene

The temperature dependence of monolayer graphene resistance is mainly attributed to the different scattering mechanisms: Coulomb scattering [34, 35], short-range scattering [36], and phonon scattering [37, 38]. Shown in Fig. 3a is the temperature dependence of the resistance of the bilayer graphene under the application of a magnetic field 0 and 12 T, respectively. The results show that the resistance of the bilayer graphene drops following nonmetallic behavior as the temperature increases from 2 to 340 K. This implies that the bilayer graphene resistors have intrinsic semiconductor properties as mentioned earlier. This can be explained by the decrease in Coulomb scattering with temperature for bilayer graphene due to its parabolic band structure. For *B* = 12 T, a similar trend as *B* = 0 T is obtained in Fig. 3a, where the resistance decreases with increasing temperature. However, the resistance for the entire temperature range is much larger than for *B* = 0 T. This indicates that the magnetic field opens an excitonic gap in the bilayer graphene that is thermally activated due to the Coulomb interaction ion-driven electronic instabilities [29, 39].

Ripples are a common feature of cleaved graphene because it is never atomically flat, as it is placed on a substrate such as SiO_2_ in the term of nanometre-scale deformations or ripples [40, 41]. Despite the magnitude of the ripples is quite small, the effect is still responsible for the unusual transport behavior of graphene, also susceptible to adsorbed impurities, defects, and the roughness of the underlying substrate [40–43]. Alternatively, it has been shown that suspended graphene flakes are corrugated on a mesoscopic scale, with out-of-plane deformations up to 1 nm [44, 45]. The deformation is typically smaller than the Fermi wavelength *λ*_F_, and these ripples induce predominantly short-range scattering. The observed height variation shows that the surface roughness beyond the atomic level is intrinsically present in bilayer graphene. Hence, one of the interesting features of the corrugation of graphene is that it offers a new experimental opportunity to study how the corrugation-induced scattering impacts the transport properties of graphene. It is important to mention that there is a strange sharp threshold like a decrease in resistance observed above 200 K. The strong temperature dependence is inconsistent with scattering by acoustic phonons. One possible explanation is that the flexural phonons confined within the ripples between the top and bottom layer cause the scattering. The presence of the ripple effect exhibits local out-of-plane ripples [44]. Theoretical calculations [41, 46] show that the scattering rates $\frac{1}{\tau }$ for interripple flexural phonons on the two-phonon scattering process as $\frac{1}{\tau}\cong \frac{\pi {t}^{\hbox{'}2}{k}_F{a}^2}{8{v}_F}{\displaystyle {\sum}_{q\ge {q}_c}\frac{q^4}{M^2{\omega}_q^2}}\left({e}^{\beta {\omega}_q}+\frac{2\beta {\omega}_q}{1-{e}^{-2\beta {\omega}_q}}\right)\times \frac{1}{{\left({e}^{\beta {\omega}_q}-1\right)}^2}$, where *ω*_*q*_ ∝ *q* ^2^ is the flexural-phonon frequency, *t*′ the derivative of the nearest-neighbor hopping integral on deformation, *a* lattice constant, $\beta =\frac{\hslash }{T}$, and *M* the mass of carbon atom [46]. For low temperatures *T* $\left({q}_T<<\frac{2\pi }{d}\right)$, few flexural modes can be excited inside ripples (*ΔR* ≈ 0). The conductivity of the surface roughness model at the limit *n* → 0 at low temperature is ${\sigma}^R\left(n\to 0\right)\sim \frac{1}{n^2}$ [45, 46]. As the temperature increases and the typical wavelengths become shorter, short-range scattering excites the flexural phonons. For the high-temperature limit, based on the above expression, we can estimate that *ΔR* ≈ (*ℏ*/*e* ^2^)(*Td*/2*πka*)^2^, which yields ∆*R*~100 to 1000 Ω at T = 300 K. The model of quenched-ripple disorder [46] suggested that the electron scattering of the static ripples quenched from the flexural phonon disorder can mimic Coulomb scattering when at room temperature. One should also note that the model predicts stronger temperature dependence (above a certain quenching temperature of about 100 K) which is close to our experimental result at about 200 K. However, the ripple effect normally leads to a rapid increase in the *R*-*T* curve rather than the sudden decrease in *R*-*T* as observed for our bilayer graphene. In the absence of a theory to explain the stronger temperature-dependent behavior, we propose that the behavior is consistent with the ripple effect interlayer scattering instead of interlayer scattering. Figure 3b shows the schematic illustration of scattering mechanisms in bilayer graphene. For a bilayer graphene, the interlayer scattering between the top and bottom ripple graphene layer is similar to Coulomb scattering with a stronger dependence on temperature. The rapid decrease in *R*-*T* above 200 K can be attributed to the transition between the low- and high-*T* limits in the interlayer ripple effect scattering. One should note that the effect of the ripple will be screened as the number layer increasing in our previous reports [47, 48].

Conversely, it was suggested that the observed strong *T* dependence could be explained by thermally excited surface polar phonons of the SiO_2_ substrate [34–37]. The SiO_2_ optical phonons at the substrate-graphene interface induce an electric field which couples to the carriers in graphene due to it modulating the polarizability [37, 38]. However, Coulomb scattering is dominant for bilayer graphene, and the substrate surface polar phonon-induced field is to some extent screened by the additional graphene layers [38]. Recently, it has been shown that the substrate dielectric constant plays an important role in scattering in graphene. Theoretical predictions show that for dielectric constant, Coulomb scattering dominates, while for dielectric constant, short-range scattering dominates, as Coulomb scattering is more strongly screened for materials with a larger dielectric constant. In fact, our observed behavior is consistent with the theory suggesting that scattering from charged impurities is dominant in graphene.

We introduce a relaxation-time approximation and treat the unscreened Coulomb potential as ${U}_S(r)={\scriptscriptstyle \frac{eQ}{4\pi {\varepsilon}_0\varepsilon r}}$ [1, 5] where *Q* is the charge of impurities. Based on the Boltzmann transport theory, we can obtain the bilayer graphene resistivity with massless Dirac fermions (MDF) at low energies as ${\rho}_{\mathrm{bilayer}}=\frac{m^{*}}{n{e}^2\left\langle \tau \right\rangle }=\frac{4{m}^2{u}_0^2}{n^2{e}^2h}\propto {n}^{-2}$. For high temperature, *E* → *k*_*B*_ *T*, we can obtain the bilayer graphene resistivity as ${\rho}_{\mathrm{bilayer}}=\frac{Z_i^2{e}^2m{N}_{ss}}{8n\hslash {\varepsilon}_s^2{k}_BT}$ [49], where *N*_ss_ is the density of impurities per unit volume, *ε*_*s*_ is the permittivity of the semiconductor, and *Z*_*i*_ is the charge state of the impurity. This shows that the resistance of bilayer graphene limited by Coulomb scattering increases as *N*_ss_ increases and decreases with increasing temperature. Considering the above analysis, we deduce that the temperature dependence of resistance in bilayer graphene is mainly determined by Coulomb scattering. The short-range scattering is independent of temperature for bilayer graphene, as the density-of-states, the matrix element, and the screening function are all energy independent. As a result of the parabolic band structure of bilayer graphene, the energy averaging of the Coulomb scattering time can give rise to the resistivity decreasing proportionally to temperature: *ρ*_bilayer_ ∝ (*k*_*B*_ *T*)^− 1^.

Based on the above discussion, we fit the measured resistance in Fig. 3a by using the following model for bilayer graphene: *R*_bilayer_ = *R*_*C* − bilayer_ + *R*_sr ‐ bilayer_, where *R*_*C* − bilayer_ and *R*_sr ‐ bilayer_ are the resistance due to the Coulomb and short-range scatterings, respectively. Figure 3b shows the relative resistance change under the biased and unbiased magnetic field as a function of temperature, and the dotted line is the fit following the equation *R*_xx_ ∝ exp(*ΔE*/*k*_*B*_ *T*), where ∆*E* is the energy gap. The opening of the energy gap due to a potential difference between the two layers and Coulomb interactions could be a cause for this. These considerations explain qualitatively why the resistance of bilayer graphene decreases with increasing temperature. Note that the relative resistance change is a strong function of temperature. At temperatures of 2~180 and 220~250 K, the relative resistance ∆*R* strongly increases as temperature increases, indicating that an energy gap forms due to many-body correction in Landau Level. When the temperature increases to *T* > 250 K, the relative resistance ∆*R* is roughly independent of the increasing temperature; this indicates that the energy gap is mostly stable at high temperatures. On the other hand, with the temperature increase from 180 to 220 K, the relative resistance ∆*R* dependence of temperature shows a sharp decrease, which indicates that the energy gap shows an anomalous thermally activated behavior as a function of temperature.

**Fig. 4:**
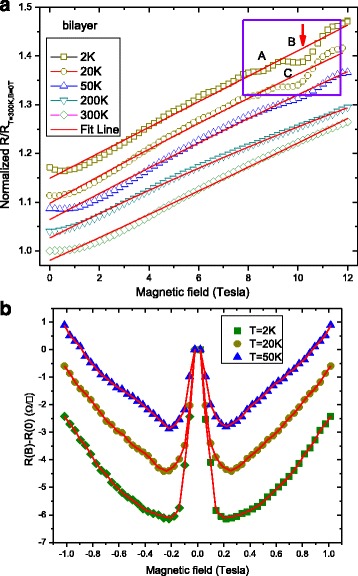
**a** Size-independent four-terminal resistance *R*_xx_ measurement of bilayer graphene as a function of magnetic field at temperature 2 K. Resistance plateau is recorded in the *R*-*H* curve. This effect originates from an augmented sublattice spin-splitting due to the high surface-impurity concentration on the graphene layer. The derivative of the resistance curve shown in the *bottom right corner inset* clearly capture the resistance plateau at ~2.5, 3.5, 4.2, 6, 7.5, 8.9, 9.5, and 11.5 T. **b** The observed four-terminal resistance nonlinear increase with a magnetic field strength of the different temperature. The *dotted line* is fitted following the reported that this gap is of excitonic nature and to increase with $\sqrt{B}$. The symbols present the measured data, and the lines are fitted

For zero gate voltage (i.e., neutrality point), we measured changes in longitudinal resistance *R*_*xx*_ as a function of applied perpendicular field *B*. Figure 4a shows the four-terminal longitudinal resistance *R*_xx_ of bilayer graphene as a function of magnetic field at *T* = 2 K at the charge-neutrality point. We have plotted the resistance per square *R*_xx_ because it is independent of a size effect of the sample. As seen from Fig. 4a, the resistance *R*_xx_ increases nonlinearly with the magnetic field strength followed by a plateau-like phase. One should note that the plateau-like phase in Fig. 4b disappears at higher temperatures. One possible explanation is the augmented sublattice spin-splitting due to the high surface-impurity concentration of the graphene layer [18]. The origin of the nonlinear magnetoresistance increment behavior is the splitting of Landau level that gives rise to a bandgap opening at the zero-energy level [31–33]. In our measurements, we fit our results to an analytical approximation for the nonlinear resistance, where *k*_*B*_ is the Boltzmann constant. We found that our results are in good agreement with this equation. These considerations explain qualitatively why the nonlinear resistance *R*_xx_ increases with the magnetic field.

**Fig. 5:**
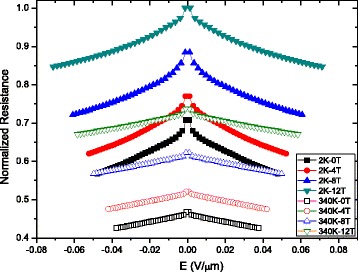
Electric- and magnetic field-dependent resistance measurements in bilayer graphene. The normalized resistance *R*-*E* curve characteristic under the magnetic field from *B* = 0 T to *B* = 12 T and the temperature from 2 to 340 K, respectively. The results show that when the magnetic field increases from 0 to 12 T, the resistance of bilayer graphene raises significantly and it decreases with the increasing electric field

Figure 5 shows the resistance of bilayer graphene as a function of the electric field (*E*) under different magnetic fields. The dependent *R*_xx_-*E* characteristics are symmetric due to the chirality of graphene electrons when an applied electric field changes from *E* to −*E*. The normalized resistance *R*_xx_-*E* curve describes the response under the applied magnetic field in the range of *B* = 0 T to *B* = 12 T and the temperatures of 2~340 K. The results demonstrate that when the magnetic field increases from 0 to 12 T at low temperatures (2~200 K) and low electric field (*E* < 0.001 V/μm), the resistance of bilayer graphene drops significantly. The larger slump in the resistance at lower temperature *T* = 2 K and low electric field as the increasing of the electric field is due to Coulomb scattering by impurities, which is a strong function of temperature. On the other hand, at high temperatures (*T* > 200 K) and electric fields (*E* > 0.01 V/μm), the resistance of bilayer graphene show a linear decrease. This can be explained by the scattering from thermally excited surface polar phonons of the *SiO*_2_ substrate being screened by the additional top graphene layers [38]. This further confirms that at high temperatures, the scattering induced by the electric field on the substrate surface polar phonons is significantly screened between top and bottom layers of bilayer graphene.

## Conclusions

In conclusion, temperature and magnetic field dependence of resistance of bilayer graphene was investigated. Intrinsic semiconductor behavior at the range of temperature 2~340 K was observed. The strange sharp threshold-like decrease in resistance around 200 K is unexpected, and we attribute it to the presence of mesoscopic ripples between the top and bottom layer. Our results reveal that the energy gap in the bilayer graphene is thermally dependent. This potentially indicates the sublattice symmetry breaking and an energy gap formation due to Landau Level splits. The obtained results are important for the better understanding of magnetic field-induced high resistance and provide indications of a theoretically predicted magnetic field-induced excitonic gap.
